# Case of Doubtful Sex

**Published:** 1847-04

**Authors:** Wm. James Barry


6. *Case of Doubtful Sex.*—In March, 1843, I was requested to examine the case of Levi Suydam, aged 23 years, a native of Salisbury, Conn. At the exciting and warmly contested election of the spring of this year, almost everything bearing the semblance of the human form, of the male sex, was brought to the ballot-box. It was at this time, and under these circumstances, that the above mentioned person was presented, by the whigs of Salisbury, to the board of selectmen, to be made a freeman; he was challenged by the opposite party on the ground that he was more a female than a male, and that, in his physical organization, he partook of both sexes.

The following was the result of the first examination. On exposing his person, I found the mons veneris covered in the usual way, an imperforate penis, subject to erections, and about two inches and a half in length, with corresponding dimensions, the dorsum of the penis connected by cuticle and cellular membrane to the pubis, leaving about one inch and a half free, or not bound up, and towards the pubic region. This penis has a well formed glans with a depression in the usual place of the meatus urinarium, a well defined prepuce, with frænum, &c. The scrotum was not fully developed, inasmuch as it was but half the usual size, and not pendulous. In the scrotum, and on the right side of the penis, one testicle, of the size of a common filbert, with spermatic cord attached. In the perineum, at the root of the corpora cavernosa, an opening through which micturition was performed, this opening was large enough to admit the introduction of an ordinary sized catheter. Having found a penis, and one testicle, though imperfectly developed, and without further examination, I gave it as my opinion, that the person in question was a male citizen, and consequently entitled to all the privileges of a freeman.

On the morning of the first Monday in April (election day) I was informed that Dr. Ticknor would oppose Suydam's admission. Suydam came forward, Dr. Ticknor objected. I then stated to the meeting, that from an examination I had made I pronounced the person in question to be a male, and requested that Dr. Ticknor, might, with the consent of Suydam, retire into an adjoining room, and examine for himself. This was done, when Dr. Ticknor stated to the meeting that he was convinced that Suydam was a male. Suydam was admitted a freeman—voted—and the whig ticket carried by one majority!

A few days after the election, it was told me that Suydam had regular catamenia. I then commenced further investigations, and learned from Mrs. Ayres, the sister of Suydam, that she had washed for him for years, and that he menstruated as regularly, but not as profusely, as most women. I next saw Suydam who very unwillingly confessed that such was the fact. I then requested him to meet Dr. Ticknor and myself the next day at my office; when the following additional particulars were elicited. Said Suydam is five feet two inches in height, light colored hair, fair complexion, with a beardless chin, and decidedly of a sanguineous temperament, narrow shoulders and broad hips, in short, every way of a feminine figure. Well developed mammæ with nipples and areola. On passing a female catheter into the opening through which micturition was performed, and through which he again stated he had a monthly, periodical, bloody discharge, instead of traversing a canal and drawing off urine, the catheter appeared to enter, immediately, a passage similar to the vagina, three or four inches in depth, and in which there was considerable play of the instrument. He stated that he had amorous desires, and that, at such times, his inclination was for the male sex; his feminine propensities, such as a fondness for gay colors, for pieces of calico, comparing them and placing them together, and an aversion for bodily labor, and an inability to perform the same, were remarked by many.

I further learned from an old lady who was present at the birth of Suydam, that, on the second day after his birth, Dr. Delamater, who attended as accoucheur, made, with an instrument, the opening through which he has since performed micturition.—Wm. James Barry, M. D., in *N. Y. Jour. of Med.*

